# Failing to Complain: Do Nursing Homes with more Residents with Dementia have Fewer Complaints?

**DOI:** 10.1093/geroni/igab046.3079

**Published:** 2021-12-17

**Authors:** Kallol Kumar Bhattacharyya, Lindsay Peterson, John Bowblis, Kathryn Hyer

**Affiliations:** 1 School of Aging Studies, University of South Florida, Tampa, Florida, United States; 2 University of South Florida, Bradenton, Florida, United States; 3 Miami University, Oxford, Ohio, United States; 4 University of South Florida, University of South Florida, Florida, United States

## Abstract

The majority of nursing home (NH) residents have Alzheimer’s Disease or Related Dementias (ADRD). However, the association of ADRD prevalence and NH quality is unclear. The objective of the current study is to understand the association of NH characteristics, including the proportion of ADRD residents, with the prevalence of NH complaints as an indicator of quality of care and quality of life. We merged data from the ASPEN Complaints/Incident Tracking System with national NH data from the Certification and Survey Provider Enhanced Reports, the Minimum Data Set, the Area Health Resource File, and zip-code level rural-urban codes in 2017. Three groups of NHs were created, including those whose proportion of residents with ADRD was in the top decile (i.e., high-dementia NHs (N=1,473)) and those whose proportion of ADRD residents was in the lowest decile (i.e., low-dementia NHs (N=1,524)). Bivariate results revealed high-ADRD NHs had higher percentages of Medicaid-paying residents, were less likely to be for-profit and chain-affiliated, had lower staffing hours and lower percentages of Black, Hispanic, and Asian residents. Using NHs in the middle deciles as reference, negative binomial regression models showed that having a low proportion of ADRD residents was significantly associated with higher numbers of total complaints (p<.001) and substantiated complaints (p<.001), whereas having a high proportion of ADRD residents was significantly associated with lower numbers of substantiated complaints (p=.001). The findings suggest the proportion of residents with ADRD in NHs is associated with quality, as measured by complaints. Policy implications of these findings will be discussed.

